# Microbial and physicochemical changes in green bell peppers treated with ultrasonic-assisted washing in combination with *Thymus vulgaris* essential oil nanocapsules

**DOI:** 10.1038/s41598-024-67358-2

**Published:** 2024-07-17

**Authors:** Saiede Akbari, Mohsen Radi, Mehdi Hosseinifarahi, Sedigheh Amiri

**Affiliations:** 1 Department of Food Science and Technology, Yasuj Branch, Islamic Azad University, Yasuj, Iran; 2 Sustainable Agriculture and Food Security Research Group, Yasuj Branch, Islamic Azad University, Yasuj, Iran; 3 Department of Horticultural Science, Yasuj Branch, Islamic Azad University, Yasuj, Iran

**Keywords:** Green bell pepper, *Thymus vulgaris* essential oil, Nanoemulsion, Peroxidase activity, Microbiology techniques, Plant physiology

## Abstract

In this study, the effect of *Thymus vulgaris* essential oil (TVO) nanoemulsion (NE, 500 mg/L) in combination with ultrasound (ultrasound-NE) on the microbial and physiological quality of green bell pepper was investigated. The TVO-NE droplet size and zeta potential were 84.26 nm and − 0.77 mV, respectively. The minimum inhibitory concentrations of the TVO and TVO-NE against *E. coli* and *S. aureus* were about 0.07 and 7 g/L, respectively. The NE-ultrasound treatment exhibited the lowest peroxidase activity and respiration rate with no detrimental effect on texture, total phenolic content, antioxidant activity, pH, and TSS. Although the NE-ultrasound treatment showed the highest weight loss and electrolytic leakage, it exhibited the best visual color and appearance. The NE-ultrasound treatment descended the total viable/mold and yeast counts significantly compared to control. Results showed that treating the bell peppers with NE-ultrasound can result in bell peppers with good postharvest quality and extended shelf life.

## Introduction

Green bell pepper (*Capsicum annuum L.*) is the horticultural product of tropical and subtropical regions. In recent years, green bell pepper has attracted a lot of attention due to its important role in the prevention of cardiovascular disease, cancer, atherosclerosis, and hemorrhage as well as slowing the process of aging. However, the limited shelf life of this product due to fungal infection, shriveling, and deterioration has affected the acceptability and marketability of this fruit. However, in recent years, different methods including hot water dipping^1^, temperature and modified atmosphere packaging^2^, UVC-treatment^3^, vacuum packaging^2^, edible coatings^4^, application of essential oils (EOs), etc. have been progressed to maintain and extend the shelf life of green bell pepper. While these methods have been effective in delaying fruit spoilage or decreasing microbial growth, they also come with their own set of challenges and limitations including damaging or affecting the taste, texture, and flavor profile of the fruits (particularly in hot water dipping), requiring specialized equipment and materials, which can be costly for producers (especially in modified atmosphere packaging) as well as they may not provide indefinite preservation^5^. On the other hand, chlorine washing and organic acids (such as acetic, citric, or lactic acid) have been widely used methods in recent years due to their effectiveness in reducing microbial load^1,6^. However, concerns about chemical residues and potential formation of harmful by-products have driven the search for safer alternatives.

Among the mentioned methods, the application of EOs as a viable solution has attracted the attention of researchers. Essential oils are the secondary metabolites of medicinal plants with the capability of inhibiting the growth of fungi, bacteria, viruses, and insects. In this regard, *Thymus vulgaris* EO (TVO) as a monoterpene EO is a good choice due to its high thymol content, which is a strong antimicrobial agent. *Thymus vulgaris* EO is considerably effective against a wide range of pathogenic bacteria^7^. However, low stability against light, temperature, during storage and processing as well as high volatility and unacceptable flavor have decreased the application of this EO. In recent years, nanoemulsions (NEs) have been used extensively to encapsulate bioactive compounds (such as EOs) to overcome their usage challenges^8^. It has been reported that NEs improve the antimicrobial properties of EOs, decrease their pungent flavor, and increase the possibility of their usage in foodstuffs^9^. Nanoemulsions are unstable thermodynamically colloidal dispersions that consist of two dispersed phases with droplets smaller than 200 nm and a continuous phase^10–12^. Many reports have encapsulated bioactive compounds by micro- or nanoemulsions including the production of cinnamaldehyde nanoemulsion^9^, *Thymus vulgaris* EO microemulsion^13^, and thymol-NE^14^. Robledo et al.^15^ reported that thymol-NE showed good antifungal activity against tomato fungi in tomato. Xu et al.^16^ reduced the apple juice enzymatic browning by using cinnamon EO-NE accompanied with ascorbic acid.

Ultrasound is a process that can break the intermolecular bonds. The sound waves with high energy transmit through an aqueous medium and subsequently produce some chemical and physical effects including cavitation, stirring, and high shear forces. Ultrasound-assisted washing involves the use of ultrasonic waves to enhance the cleaning efficiency of washing treatments. It works by generating cavitation bubbles that implode, creating localized high-pressure zones that disrupt microbial cells and remove surface contaminants more effectively than conventional washing. Ultrasound is an economical and simple-to-use technology that currently has been used in emulsification, non-destructive tests, homogenization, and other fields of food processing and storage^17^.

However, due to the limited effect of ultrasound in its individual use (on microbial inactivation which needs usually a long time for good effectivity), the researchers prefer to study on ultrasonic-assisted chemical disinfectants or antimicrobial agents. The use of ultrasound in combination with cinnamon EO fumigation was found to be effective in improving the quality of fresh-cut asparagus^18^. He et al.^19^ also reported that the ultrasound-treated curcumin/orange EO nanoemulsions improved the quality of cherry tomato. Casco et al.^20^ evaluated the effect of ultrasound treatment in combination with green tea extract, nisin, or natamycin on the main spoilage factors and some quality indicators of a mixed fruit and vegetable smoothie. Millan-Sango et al.^21^ combined ultrasound treatment with thyme and oregano EOs to reduce the microbial population on lettuce leaves. While some studies have investigated the effects of EOs in combination with ultrasound, there is a lack of research on the combined application of ultrasound and NEs as two separate processing steps. In contrast, some studies have used ultrasound to form NEs, which are then applied to fruits and vegetables for bacterial inactivation. However, the combined use of ultrasound and NEs has not been thoroughly evaluated, and the use of TVO-NE in this context warrants special attention. On the other hand, due to challenges that still exist for the preservation of green bell peppers, we aimed to evaluate the simultaneous effect of *Thymus vulgaris* EO-NE and ultrasound on shelf life and postharvest quality of green bell pepper fruits during cold storage.

## Materials and methods

### Materials

*Thymus vulgaris* EO (TVO) was obtained from Gol-Ghatreh (Fars, Iran). Phenolphthalein, Tween80 (T80), NaOH, Ba(Cl)_2_, oxalic acid, ascorbic acid, metaphosphoric acid, and all other chemical reagents were obtained from Merck (Darmstadt, Germany). *Staphylococcus aureus* (ATCC 25923) and *Escherichia coli* (ATCC 25922) were purchased from the Persian Type Culture Collection (PTCC).

### Preparation of TVO nanoemulsion

A coarse emulsion of TVO (500 mg), distilled water (1000 mL), and T80 (20 g) was prepared by stirring at 1200 rpm for 10 min. After the formation of the coarse emulsion, ultrasonication (Q700, Q-Sonica, USA) was performed (100 W cm^−2^, 4 min) until a clear NE (500 mg/L) was obtained^9^. The produced NE was stable for 3 months.

### Particle size analysis of NE

The particle size of the TVO-NE was determined with a Nano ZS instrument (5.02 version, Malvern Instruments Ltd., UK) equipped with a 4 mW He–Ne laser and a detection angle of 173° with a wavelength of 633 nm. The dynamic viscosity was 8.76 mPa s (at 25 °C). The results were recorded as the z-average, hydrodynamic droplet size, and polydispersity index^22^.

### Antimicrobial activity

#### The minimum inhibitory concentration (MIC) and minimum bactericidal concentration (MBC)

To determine the MIC and MBC of the free TVO and TVO-NE, the broth dilution method was used against *S. aureus* and *E. coli*. For this purpose, 2 mL of nutrient broth was added to sterile tubes containing 0.001 to 0.7% of free or nanoemulsified TVO. Thereafter, 50 μL of each tested bacterium containing 10^6^ CFU/mL was inoculated. The tubes were incubated at 37 ± 0.1 °C for 24 h, bacterial growth was evaluated for 3 days, and the concentration in the tube at the lowest antimicrobial concentration with no visible growth was considered as the MIC^23^.

To measure MBC, 2 mL of nutrient broth was mixed with 100 μL of the MIC tube content which no microbial growth was observed, and then incubated at 37 ± 0.1 °C for 24 h. The lowest concentration of free TVO and TVO-NE, in which no microbial growth occurred, was selected as the MBC^7^.

#### Agar diffusion method

The thymol and its NE antimicrobial activity was evaluated using the agar well diffusion method^23^. Thereby, VRBA (for *E. coli)* and BPA (for *S. aureus*) wells were inoculated with 0.1 mL of *S. aureus* and *E. coli* suspensions (containing 10^6^ CFU/mL) and 50 μL of TVO solutions (10 g/L, in the free and NE forms). The inhibition zones were measured after the incubation of plates at 37 ± 2 °C for 24 to 48 h.

### Preparation of green bell pepper treatments

Green bell pepper fruits, uniform in size and ripeness stage, with no infection or physical injury, were harvested from a local orchard in Yasuj. The bell peppers were cut into big pieces and then divided into six parts to prepare the treatments (Table 1). Two groups of green bell peppers were sonicated (Q700, Q-Sonica, USA) in water (ultrasound-water) and in NE (ultrasound-NE) for 10 min to prepare the ultrasound-water and ultrasound-NE treatments. The third group of treatments was submerged in NE for 10 min without being sonicated (NE). In another treatment, the bell peppers were just dipped in water for 10 min (control-water). A treatment was prepared by submerging the bell peppers in benzalkonium chloride as a commercial disinfecting agent for 10 min (benzalkonium chloride sample) and a control sample with no treatment was also considered. After treating all samples, the fruits were let dry at the ambient condition and were packed in polyethylene bags, and stored for 10 days at 5 ± 1 °C. The sampling for quality evaluation was performed at 0, 1, 4, 7, and 10 days of storage.

**Table 1:** The research treatments.

Treatments	Description
Control	No treatment
Ultrasound-water	Sonicated in water for 10 min
Ultrasound-NE	Sonicated in NE for 10 min
Control-water	Dipped in water for 10 min
NE	Dipped in NE for 10 min
Benzalkonium chloride	Dipped in benzalkonium chloride for 10 min

### Weight loss

The weight loss of treatments was calculated by weighing them at day 0 and after 1, 4, 7, and 10 days of storage at 4 °C. Results were expressed as the percentage of weight loss relative to the initial value^6^.

### Determination of pH and total soluble solids

A digital pH meter (MA235, Mettler-Toledo Inc., Switzerland) and a refractometer (Abe, NAR-3 T, Japan) were used to measure pH and total soluble solids (TSS) at 20 °C, respectively^6^. The fruit pulp was used for the measurements.

### Peroxidase activity

To measure the peroxidase activity, a solution of guaiacol (10 mM, as substrate) and H_2_O_2_ (10 mM) in sodium acetate (50 mM) with a pH of 5.0 was prepared. The prepared solution was mixed with the bell pepper extract in a ratio of 1:1 (v/v). The change in absorbance at 470 nm µg^−1^ of total protein was expressed as the enzyme activity^19^.

### Respiration

To measure the respiration, the samples were put in a hermetically sealed container (1 L). After an hour, the respiration rate was measured by quantifying the headspace gas with an autogas analyzer (model: Checkmate 9900 O_2_/CO_2_, Dansensor, Denmark) at 25 °C. The respiration rate was expressed as mL CO^2^ kg^–1^ h^–1^^18^.

### Fruit firmness

The fruit firmness was measured using a fruit texture analyzer (CT-3 Model, Brookfield Company, USA). Thereby, the maximum force (mN) consumed versus compression was measured at a puncture depth of 3 mm, a probe speed of 1 mm/s, and a probe diameter of 30 mm^24^.

### Total phenolic content (TPC)

Total phenolic content was determined based on a spectrophotometric method (at 765 nm) according to Abdipour et al. (2020) method by using Folin-Ciocalteu reagent (FCR). Results were expressed as mg of gallic acid (GAE) per kg of the orange fruit (mg kg^−1^ GAE) based on a calibration curve constructed by plotting the serial concentrations of GAE versus absorbance^6^.

### Total flavonoid content

To measure the total flavonoid content, a colorimetric method was used at 510 nm. For this purpose, a solution of green bell pepper extract (1 µL), distilled water (4 mL), and sodium nitrite solution (5%, 0.3 mL) was prepared. After 5 min, Al-chloride (0.3 mL, 10%) was added and then after 6 min, Na-hydroxide solution (2 mL, 1 M) was added, followed by an immediate addition of distilled water (3.3 mL). The calibration curve was constructed using catechin as the standard and the total flavonoid content was reported as mg catechin equivalents per kg of sample (mg/kg)^25^.

### DPPH radical scavenging activity

The total antioxidant activity was measured according to the DPPH radical (2, 2-diphenyl-1-picrylhydrazyl) scavenging capacity method. A total of 24 mg of DPPH were dissolved in 100 mL of methanol to make the stock solution. Filtration of DPPH stock solution using methanol yielded a usable mixture with an absorbance of around 0.973 at 517 nm. In a test tube, 3 mL DPPH workable solutions were combined with 100 µL of leaf extract. Three milliliters of solution containing DPPH in 100 µL of methanol is often given as a standard. After that, the tubes were kept in complete darkness for 30 min. The absorbance was therefore determined at 517 nm. The following formula was used to calculate the percentage of antioxidants:

1 $${\text{Antioxidant}}\,{\text{activity}}\,\left( \% \right) = \left[ {\left( {{\text{Ac}} - {\text{As}}} \right) \div {\text{Ac}}} \right] \times {1}00$$

where Ac is the control reaction absorbance and As is the testing specimen absorbance^26^.

### Electrolyte leakage

For measuring electrolyte leakage, 2 g of bell pepper tissue was placed into 90 mL of deionized water and incubated at 25 ºC for 60 min. Before (EC_1_) and after (EC_60_) incubation, the solution electrical conductivity (Mettler Toledo LE703) was measured. Then the solution was boiled (121 ºC-25 min) and again the EC, after boiling (EC_T_), was measured. Electrolyte leakage was measured according to the following Eq. ^27^.

2 $${\text{Electrolyte}}\,{\text{leakage}}\,\left( \% \right) = ({\text{EC}}_{{{6}0}} - {\text{EC}}_{{1}} )/{\text{EC}}_{{\text{T}}} \times {1}00$$

### Microbiological analysis

The bell pepper sample (10 g) and sterile saline (90 mL) were mixed in a Stomacher® for 4 min. The proper serial dilutions were then surface-plated onto PCA and YGC at 37 °C for 48 h and 7 d, respectively, to determine the total viable count and total mold and yeast. The bacterial counts were reported as log CFU/g of sample^28^.

### Yellowing index

The yellowing index was calculated by the equation below^18^:

3 $${\text{Yellowing}}\,{\text{index}} = \left( {{\text{A}} \times {\text{B}}/{\text{C}} \times {\text{D}}} \right) \times {1}00\%$$

where A is the single plant yellowing grade; B is the number of plants at this grade; C is the maximum yellowing grade; D is the total number of plants. There are five grades in the bell pepper spears: 0 = no yellowing area; 1 = approximately 1–25% yellowing area; 2 = approximately 26–50% yellowing area; grade 3 = approximately 51–75% yellowing area, and 4 = more than 75% yellowing area.

### Statistical analysis

The experiments were performed in triplicate. The data were reported as the mean ± standard error. The one-way analysis of variance with differences at *P* < 0.05 was used for the data analysis.

## Results and discussion

### Particle size analysis of *Thymus* vulgaris EO-NE

**Figure 1:**
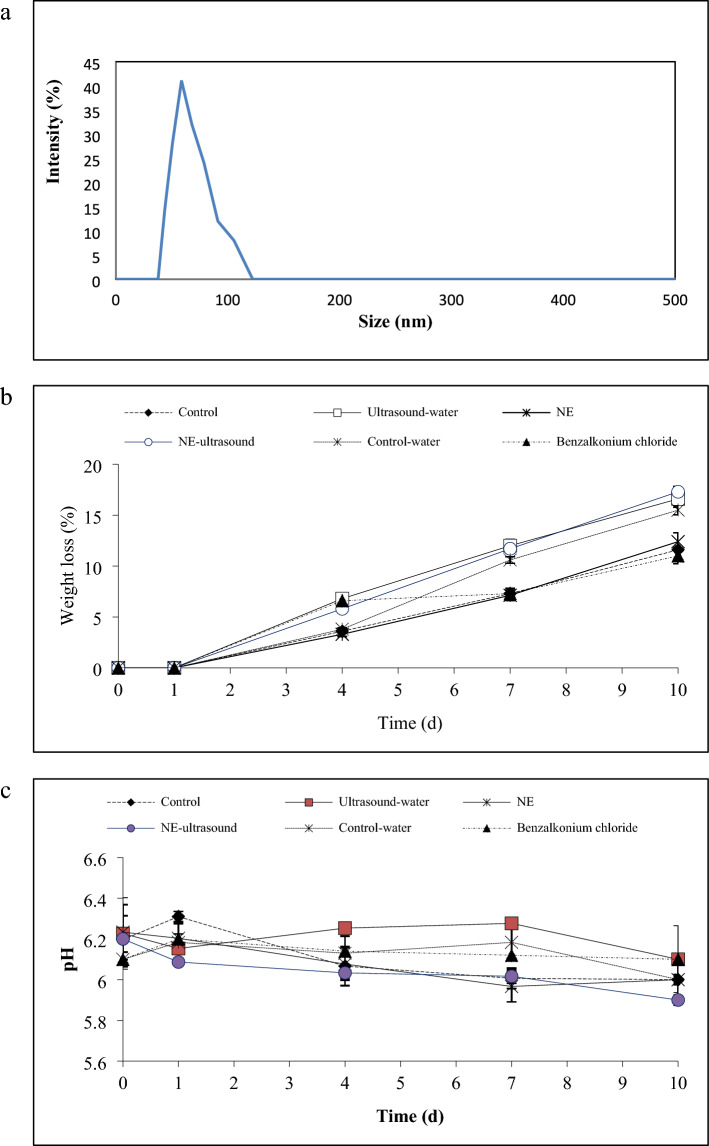

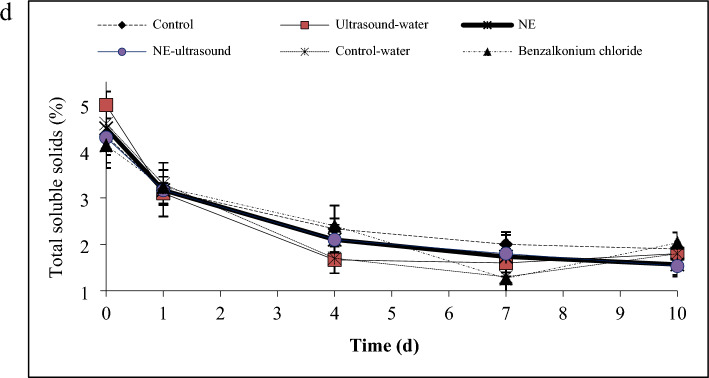
The particle size distribution of Thymus vulgaris essential oil-NE; (**a**); weight loss (**b**), pH (**c**), and total soluble solids (**d**) of different green bell pepper treatments during cold storage.

The TVO particle size distribution is depicted in Fig. 1a. The particle size distribution of the NE was 84.26 nm. According to Fig. 1, one narrow peak with a low polydispersity index (0.25) was detected for the NE sample. The low polydispersity index was an indication of good homogeneity of the system. The NE zeta potential was − 0.77 mV, confirming the stability of the droplets due to the repulsive forces between them^29^. da Silva et al.^30^ formulated a *Origanum vulgare* EO-NE with droplet size ranging from 41.67 to 231.83 nm. Similar results were also obtained by Karimi-Khorrami et al.^14^ on thymol-NLC, Sepahvand et al.^8^ on thymol-NE, Salvia-Trujillo et al.^31^ on lemongrass EO-Al NE, Almasi et al.^7^ on thyme oil-microemulsion, and Hojati et al.^9^ on cinnamaldehyde-NE.

### MIC and MBC of *Thymus* vulgaris EO-NE

The TVO-NE showed a MIC value of 7 g/L against *E. coli* and *S. aureus,* and a MBC value of 7 g/L against the two bacteria (Table 2). However, free TVO showed lower MIC (0.07 g/L) and MBC (0.2 g/L) values against *E. coli,* and* S. aureus*. The MIC and MBC values of free TVO increased significantly in the NE system which may be due to the extremely small droplet sizes of the NE, which facilitates their penetration into microbial cells, disrupting the cell membrane integrity, and causing leakage of cellular contents. Meanwhile, nanoemulsification helps to solubilize the EOs in water, increasing their bioavailability, and allowing better interaction with microbial cells. This improved bioavailability contributes to the reduction in MIC^32^. Ma et al.^33^ also reported that thyme oil-microemulsion showed a higher MIC value than that of the free oil.

**Table 2:** The MIC and MBC of TVO in free and nanoemulsified forms against *E. coli* and *S. aureus*.

Samples	*E. coli*		*S. aureus*	
	MIC (g/L)	MBC (g/L)	MIC (g/L)	MBC (g/L)
Free TVO	0.07 ± 0.00^a^	0.20 ± 0.00^a^	0.07 ± 0.00^a^	0.30 ± 0.00^a^
NE	7.00 ± 0.07^d^	10.00 ± 0.00^d^	7.00 ± 0.14^d^	10.00 ± 0.70^d^

*Each point is the average of three replicates, and values with similar letters in each column are not significantly different (P ≥ 0.05).

#### Agar diffusion method

Free EO showed greater inhibition zones than that of TVO-NE (Table 3) against the two tested bacteria. The reason for this better function is not discussed by the researchers. However, in the agar diffusion test, the diffusion of the antimicrobial agent is important for its function against the bacteria, and it seems that TVO and TVO-NE could diffuse in the culture media in the same way, and the higher concentration of free TVO resulted in better performance of free TVO than that of TVO-NE against *E. coli* and *S. aureus.* These results were in line with Sepahvand et al.^8^ results who reported a higher inhibition zone for free thymol compared to thymol-NE against *S. aureus* and Viyoch et al.^34^, who confirmed that the pure basil oil inhibition zone was greater than that of microemulsified basil oil.

**Table 3:** Antimicrobial activity of TVO in free and nanoemulsified forms against *E. coli* and *S. aureus*.

Samples	*E. coli*	*S. aureus*
	Inhibition zone (mm)	
Free TVO	50.00 ± 1.15^a^	40.00 ± 0.91^a^
NE	13.00 ± 0.10^b^	11.00 ± 0.50^b^

*Each point is the average of three replicates, and values with similar letters in each column are not significantly different (P ≥ 0.05).

### Weight loss

Fruits and vegetables weight loss occurs as a result of carbohydrate consumption and water loss through respiration and transpiration processes, resulting in texture shriveling^35^.

Figure 1b shows bell peppers weight loss during the storage time. The weight loss of all samples increased during the storage. In this regard, the samples treated with ultrasound (Ultrasound-Water and NE-Ultrasound) indicated the highest (p < 0.05) weight loss (about 17.0% after 10 days of storage) followed by the control-water sample (15.5% on day 10). However, the NE and control samples revealed the lowest weight loss (p < 0.05) over time (12.0% after 10 days). The Benzalkonium chloride, aligned with the ultrasound-treated samples, showed a fast weight loss until the day 4th. After day 4, the weight loss decreased and followed the trends of NE and control.

The highest weight loss in ultrasound-treated samples could be attributed to the effect of ultrasound vibrations on the promotion of fruit cell wall permeability. This may have allowed water molecules to more easily pass through the cell membranes. However, treating the fruits and vegetables with water alone does not appear to be an effective approach, as it increased the weight loss in the bell pepper fruit, which can lead to increased fruit decay and deterioration.

The TVO-NE treatment did not have a significant effect on fruit weight loss, as the trend was similar to the control. The cinnamon EO-treated tangerine weight loss was not also different from the control in the Radi et al.^32^ study. Contrary to our results, Wang et al.^18^ showed that the weight loss of ultrasound + cinnamon EO-treated fresh-cut asparagus was lower than the control sample after 20 days of storage.

### pH and total soluble solids

The results of pH and TSS are shown in Fig. 1c,d. The fruit pH and acidity affect their acceptability and very high or low pH is not suggested for qualified fruits^36,37^. The pH values of bell peppers (Fig. 1c) remained stable at 6.1 across all samples throughout the storage period, indicating consistent pH maintenance.

A gradual and statistically significant (p < 0.05) decline in TSS was observed in all bell pepper fruits, decreasing from 4.5% to 1.8% (Fig. 1d). However, there was no notable difference in TSS reduction among the various samples (p ≥ 0.05). TSS decrease may be due to the increase in the respiration rate of the fruits (according to the results of respiration). The respiration process leads to the loss of organic acids and the breakdown of sugars, resulting in a decrease in TSS. Moreover, the enzymatic activity of the fruits during storage breaks down complex carbohydrates into simpler sugars and further converts these sugars into other compounds. This process ultimately leads to a reduction in TSS levels^6,38^.

Ranasinghe et al.^39^ reported that the application of cinnamon extract did not influence the TSS, total acidity, and pH of bananas over 21 days of storage. Perdones et al.^40^ declared that lemon EO-chitosan coating did not show any significant effect on strawberry pH, acidity, and TSS over storage. In addition, Akhavan et al.^28^ reported that treating the date palm fruit with cinnamaldehyde-loaded nanostructured lipid careers decreased pH and TSS in comparison with the control. Wang et al.^18^ showed that the TSS of their fresh-cut asparagus treatments decreased over time and the ultrasonic-assisted citric acid and nisin washing + cinnamon EO fumigation treatment significantly delayed the decrease in TSS of the fresh-cut asparagus.

### Peroxidase activity

**Figure 2:**
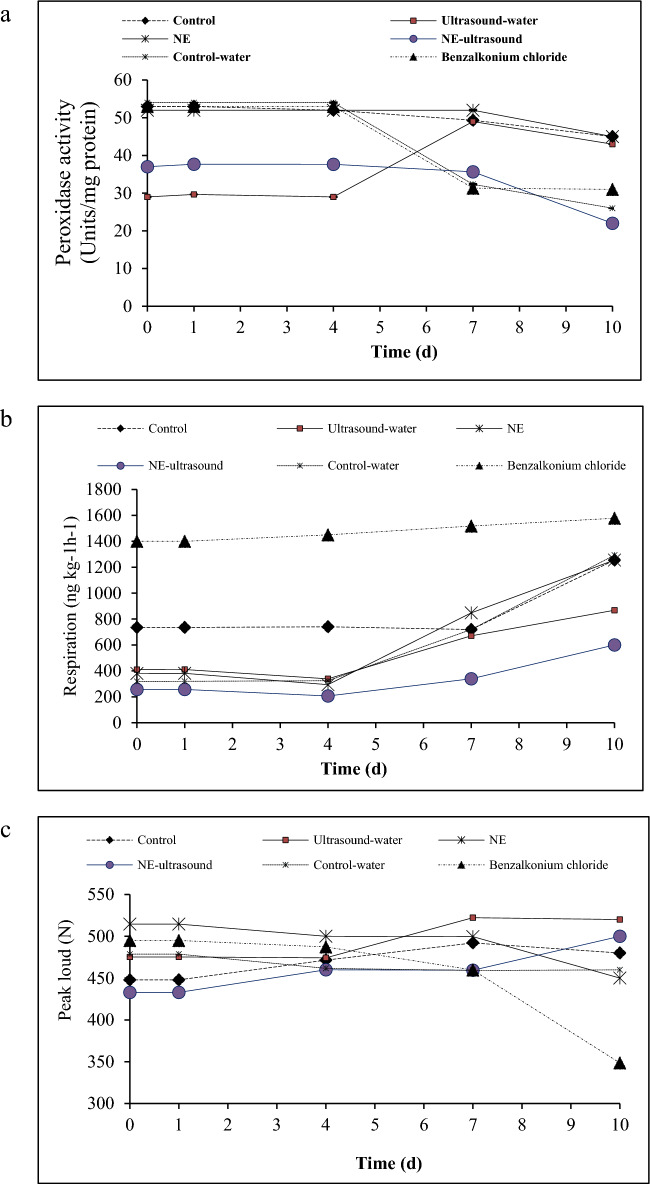
Peroxidase activity (**a**), respiration rate (**b**), and texture (**c**) of different green bell pepper treatments during cold storage.

Analysis of peroxidase activity (Fig. 2a) revealed that the control, NE, benzalkonium chloride, and control-water samples exhibited the highest enzyme activity at day 0. However, this enzyme activity remained constant in the control and NE until day 7 and then decreased slightly from 49 to 44 unit/mg protein until day 10. But in the benzalkonium chloride and control-water samples, a significant decrease in the enzyme activity from 53.5 to 31.5 unit/mg protein occurred from day 4th to 7th and remained constant from day 7 to 10, indicating the efficiency of these treatments in the activity reduction of peroxidase with a delay of four days.

In the NE-ultrasound and ultrasound-water samples, the peroxidase activity decreased significantly from the first day of storage, indicating the efficiency of these treatments in the reduction of enzyme activity. In this regard, the NE-ultrasound sample was the preferred sample as this sample kept the enzyme activity at a low level of 35.6 unit/mg protein until day 7, and after that, the enzyme activity decreased to a lower amount of 22 unit/mg protein. But in the ultrasound-water sample, a significant increase in the peroxidase activity was observed from the 4th to the 7th day of storage as it achieved the 49 unit/mg protein value (similar to the control sample) and it follows the same trend as that of the control sample until the end of the storage time.

Peroxidases are a group of enzymes that catalyze the oxidation of various substrates using hydrogen peroxide. Higher levels of peroxidase activity can lead to an increase in the browning process and tissue softening. The reduction in peroxidase activity indicates a delay in the ripening process and a delay in senescence. Consequently, regulating peroxidase activity during fruit storage is crucial for preserving fruit quality and prolonging shelf life^41^. According to the results, benzalkonium chloride, control-water, and ultrasound-water were effective in the reduction of peroxidase activity. In this regard, NE-Ultrasound showed the best efficiency.

The higher inhibitory effect of NE-Ultrasound may be explained by the fact that ultrasound promoted the penetration of TVO-NE into fresh-cut bell pepper, which further facilitated the direct interaction of the bioactive compound with the active site of peroxidase enzymes, competitive inhibition of the bioactive compound with the peroxidase substrates, or binding of the bioactive compound to allosteric sites on the enzyme molecule, thereby, preventing the normal function of the enzyme^18^.

Das et al.^42^ reported that the application of *Myristica fragrans* EO-loaded chitosan NE in stored rice reduced the lipid peroxidation caused by lipases and peroxidases, indicating the reduction of these enzymes activity. Wang et al.^41^ indicated that the lowest peroxidase activity was detected in the clove EO nanoemulsions-coated Fresh *Tremella fuciformis* even on the 9th day of storage. Wang et al.^18^ showed that ultrasonic-assisted citric acid and nisin washing combined with cinnamon EO fumigation retarded the activity of peroxidase. Das et al.^42^ stated that the application of *Angelica archangelica* EO-chitosan nanoemulsion in table grape coating decreased the activity of peroxidase, which was greater than the control. Ding et al.^43^ reported that the activities of the disease resistance enzymes (like peroxidase) enhanced in blueberry fruit treated with thymol.

### Respiration

The respiration of different bell pepper fruit treatments is shown in Fig. 2b. The respiration increased in all samples over time. Surprisingly, benzalkonium chloride sample showed the highest respiration rate throughout the storage time, which increased from 735 to 1011 ng kg^−1^ h^−1^ during the 10 days of storage. After this sample, the control sample showed the highest respiration. After the control, ultrasound-water, control-water, and NE were placed and no significant differences were detected among these samples until the 7th day. After day 7, the ultrasound-water sample respiration decreased to a lower value than the NE and control-water. From the 7th day onwards, the control, NE, and control-water samples showed similar respiration values. The lowest respiration values were observed in the NE-ultrasound sample.

Therefore, the use of NE, ultrasound-water, and control-water treatments was effective in the reduction of the fruit respiration before the 7th day. But treating the bell peppers with NE-ultrasound gave the best result. This can be due to the effect of ultrasound on the cell membrane permeability improvement, which in turn enhances the absorption of TVO-NE into the fruit texture. The gradual EO release from the NE into the fruit cells improved the function of EO in decelerating the cellular biochemical/physiological processes such as ethylene production through inhibiting transcription of related genes and reducing respiratory rates^18^.

Similar results were obtained by Azarakhsh et al.^44^ in coating fresh-cut pineapple with lemongrass essential EO-loaded alginate solution and Das et al.^42^ in the usage of *Angelica archangelica* EO*-*loaded chitosan nanoemulsion on table grape fruit. Wang et al.^18^ declared that ultrasonic-assisted citric acid/nisin washing + cinnamon EO treatment reduced respiration rate and delayed respiration peak time in fresh-cut asparagus.

### Texture

As shown in Fig. 2c, the bell peppers hardness remained unchanged over time for all samples and also no significant difference was observed among the various treatments during the 10-day storage period. This indicates the compatibility of the treatments with the fresh-cut bell pepper fruit. The only exception was the benzalkonium chloride treatment, which its hardness began to decrease from day 7 onwards.

Firmness is a significant parameter in increasing fruit shelf life and marketing. Firmness reduction occurred during the ripening process which is due to the activities of cell wall hydrolyzing enzymes and the conversion of protopectin into pectin^6^. According to the results, benzalkonium chloride increased and accelerated the respiration rate, therefore might increase the ethylene production and activate the hydrolytic enzymes (like cellulase, polygalacturonase, and xylanase) and hence, speed the trend of bell pepper softening.

Wang et al.^18^ and He et al.^19^ declared that the ultrasonic + cinnamon EO fumigation and ultrasound-thyme EO NE treatments, respectively, did not show any negative impact on texture compared to the control.

### Total phenolic content

**Figure 3:**
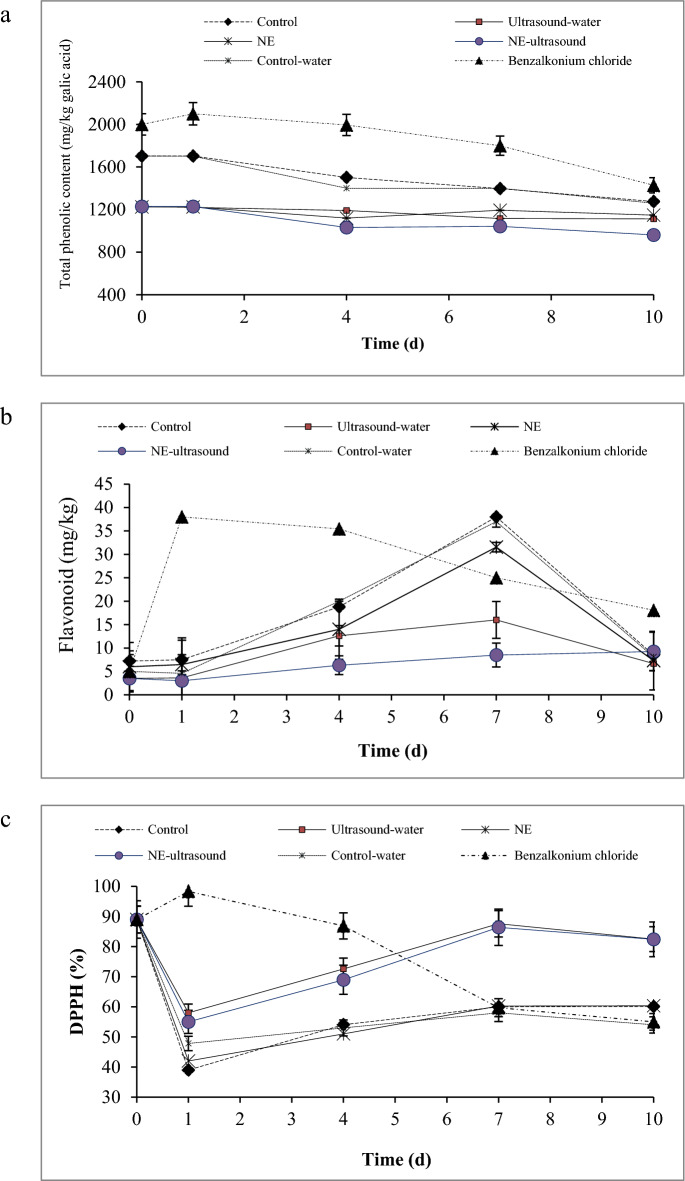
Total phenolic content (**a**), flavonoid content (**b**), and DPPH (**c**) radical scavenging activity of different green bell pepper treatments during cold storage.

Phenolic compounds are produced by plants as secondary metabolites. These compounds have significant roles in plants flavor, color, and quality^10^. Figure 3a displays the TPC of green bell pepper fruits. The TPC of all fresh-cut bell peppers gradually decreased with prolonged storage time. The TPC of the benzalkonium chloride and control/control-water samples were the highest, respectively, throughout the storage time, followed by the NE and water-ultrasound samples. In addition, the NE-ultrasound treated sample exhibited the lowest TPC throughout the storage period. The above results revealed that the NE, water-ultrasound, and NE-ultrasound treatments inhibited the accumulation of TPC in the fresh-cut bell pepper fruit, and the NE-ultrasound treatment had the lowest TPC.

Phenylalanine ammonia lyase and peroxidase are crucial enzymes in the phenolic metabolic pathway. Furthermore, the inhibitory effect of NE-ultrasound was higher than that of application of NE or ultrasound, solely. This may be explained by the fact that ultrasound promoted the penetration of NE into fresh-cut bell pepper, leading to a decrease in peroxidase and phenylalanine ammonia lyase activities. According to previous studies, EOs can reduce the activity of these enzymes during fruit storage by virtue of their volatile compounds, particularly phenolic derivatives, which effectively inhibit phenylalanine ammonia lyase and peroxidase activities^45^. Das et al.^42^ and Radi et al.^32^ obtained similar results on using *Angelica archangelica* EO-chitosan-NE on table grape fruit and cinnamon EO nanostructured lipid carriers on tangerine, respectively. Ochoa-Velasco and Guerrero-Beltrán^46^ reported that the TPC of white prickly pears was not affected by chitosan coating containing acetic acid. Meanwhile, in rowanberry, no significant change occurred in TPC before 16 days, but it decreased after this time^47^. It has been reported that, in citrus products, the conversion of phenolic compounds resulted in a taste change from bitter to sweet. Meanwhile, reports confirm the impact of phenolic compounds on the astringent taste of stored and processed carrots^47^. In line with our results, Wang et al.^18^ reported that treated fresh-cut asparagus with ultrasonic-assisted citric acid and nisin washing (CNUS) combined with cinnamon EO fumigation showed the lowest TPC during the storage time.

### Flavonoid content

Flavonoids are a group of phenolic compounds that are widely distributed in fruits and vegetables. They show significant roles in plant development, growth, and defense against different tensions as well as contribute to the color, taste, and aroma of fruits. The flavonoid content of all samples increased significantly at first and then decreased until the end of storage (Fig. 3b). In less ripened or immature green bell peppers, flavonoids are synthesized through enzymatic reactions and therefore the flavonoid content increased. Consequently, these bell peppers have higher levels of certain flavonoids compared to fully ripe ones. Hence, the decrease of flavonoid content in the second stage of storage was due to the fruits ripening process, which leads to the degradation of flavonoids by means of enzymatic reactions including hydrolysis or oxidation^47^.

The increase in flavonoid content of the benzalkonium chloride sample (until day 1) was much faster and, of course, higher (38 mg/kg) than the other samples. This sample entered the ripening process much faster (on day 1) than the other samples due to the highest rate of respiration, resulting in a significant reduction in the flavonoid content after day 1.

After the benzalkonium chloride sample, the control and water-control samples showed the highest flavonoid content while maintaining the increasing/decreasing trend. In these samples, the increasing trend of flavonoid production lasted until the 7th day, indicating that the ripening process was delayed to day 7. As ultrasound was not used in these treatments, the enzymes involved in flavonoid synthesis and degradation were most active, resulting in a higher rate of flavonoid synthesis and degradation. However, in NE and ultrasound-treated samples like NE-ultrasound and ultrasound-water, the lowest rates of flavonoid increase or decrease were observed due to the effect of TVO-NE and ultrasound on the reduction of enzymes activities involved in the synthesis and degradation of flavonoids. The results indicated that a combination of NE with ultrasonication could suppress the synthesis of flavonoids more effectively than NE or ultrasound alone, which comes from the increase in cell wall permeability as a result of sonication, better penetration of TVO nanoparticles due to their extremely small particle size, and slowing down the ripening process^45^. BaltacioĞLu et al.^47^ evaluated the change in the flavonoid composition of rowanberry and indicated a significant reduction in rutin, quercetin-3-glucoside, and quercetin-3-D-galactoside over time. Miao et al.^48^ discovered that the irradiation of *Scutellaria baicalensis* roots with UV-A led to a flavonoid content increase over time.

### DPPH radical scavenging activity

The measurement of the antioxidant activity of green bell peppers was performed by measuring the DPPH free radical scavenging activity (Fig. 3c).

In line with the TPC results, the antioxidant activity of benzalkonium chloride treatment was the highest during the first 5 days of storage time, and after that, it decreased to the minimum level. In line with total phenolic content, the bell peppers antioxidant activity was constant and did not change during the storage time. In this regard, there was one exception including the benzalkonium chloride treatment. This treatment showed the lowest antioxidant activity, which occurred from the first day (a decrease from 89 to 58%) for the control-water sample, and from the 7 day to the same value (58%) for the benzalkonium chloride treatment.

The reduction in phenolic content and an increase in flavonoid content resulted in a constant antioxidant activity of the bell pepper samples over storage. On the other hand, the samples with constant peroxidase activity showed constant antioxidant activity. Peroxidase activity and antioxidant activity are closely related. Peroxidases play an important role in breaking down hydrogen peroxide and reactive oxygen species (ROS) into oxygen and water, therefore, preventing ROS accumulation and cell oxidative damage. On the other hand, antioxidants scavenge/neutralize ROS or support the antioxidant enzymes activity such as peroxidases, thereby inhibiting the cell oxidative stress^49^.

However, Radi et al.^32^ reported that using *thymus vulgaris* EO-loaded nanostructured lipid carriers/alginate coating increased the tangerine fruit antioxidant activity. It has been also reported that chitosan coating preserved the sweet cherry DPPH radical scavenging activity^50^. Thereby, samples with lower peroxidase activity showed lower antioxidant activity. Results showed that treating the bell peppers with water and the commercial disinfecting agent cannot be considered proper treatments regarding the antioxidant activity.

### Electrolyte leakage

**Figure 4:**
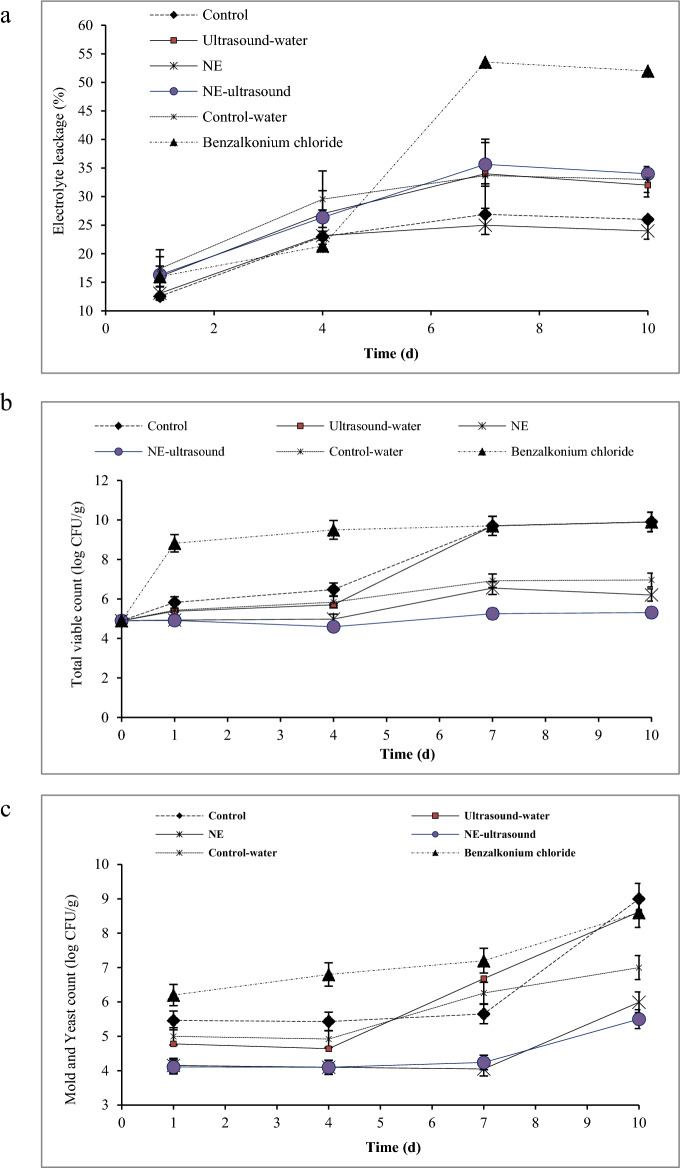
Electrolyte leakage (**a**), total viable count (**b**), and total mold and yeast count (**c**) of different green bell pepper treatments during cold storage.

To determine the integrity of membrane and tissue in vegetables and fruits, electrolyte leakage is used. The electrolyte leakage of tissue shows the weakening of cell membrane due to the ripening^27^. The percentage of electrolytic leakage increased in all bell pepper samples during the storage (Fig. 4a), demonstrating a gradual loss of integrity in the cell membrane which might be due to the damage caused by senescence and microbial growth^41^. The bell peppers treated with benzalkonium chloride had the highest electrolytic leakage during storage (52%). This sample showed a softer texture than the other samples at the end of the storage time (according to the texture analysis test) as well as it had the highest microbial population according to the microbial experiments results. The samples with no ultrasound treatment including the control and NE samples showed significantly lower leakage values (24%) than the samples treated with ultrasound (ultrasound-water and NE-ultrasound) as well as the control-water sample (32%), indicating the cell membrane damage due to the ultrasound treatment. However, the cell membrane damage and senescence were remarkability higher in the samples treated with benzalkonium chloride than the ultrasound-treated samples. It has been reported that the packaging of buckwheat microgreens in the films with 16.6 pmol/(m^2^ s Pa) oxygen transmission rate had a fresh appearance with the lowest tissue electrolyte leakage^51^. Despite the NE-ultrasound samples exhibiting higher electrolytic leakage compared to other samples, they demonstrated superior freshness during storage. This could be attributed to the enhanced penetration of TVO-NE into the ultrasound-treated texture. Additionally, the positive impact of NE on retarding physicochemical reactions within the tissue, decreasing the peroxidase activity (a crucial factor contributing to browning, discoloration, and deterioration of plant tissues), and reducing the activity of tissue-hydrolyzing enzymes, which influence fruit decay, may have contributed to the improved freshness^45^. Aday^27^ reported that the enzymatic browning of fruits is not only related to enzymes but also membrane damage. Alenyorege et al.^52^ reported that prolonged ultrasound treatment caused structural changes in cabbage tissue, resulting in increased ionic movement. In contrast to our results, Wang et al.^18^ declared the lowest electrolyte leakage in ultrasonic-assisted citric acid and nisin washing + cinnamon EO treated fresh-cut asparagus.

### Microbiological analysis

Figure 4b,c shows the green bell peppers microbial analysis. Interestingly, the highest total viable/mold and yeast counts were detected in the benzalkonium chloride treatment. Benzalkonium chloride is used commonly as a vegetable disinfecting agent, indicating the uselessness of this commercial antimicrobial agent. After benzalkonium chloride, the control sample showed the highest population for both total mesophilic/mold and yeast counts. After control, the ultrasound-water and control-water samples exhibited the highest microbial population with no significant difference among them until the 4th day. The washing effect of water in the control-water sample and the removal of surface microbial by water might be the reason for the lower microbial count in this sample. Regarding the ultrasound-water sample, the mechanical shear forces and the cavitation phenomenon caused by sonication resulted in microbial cell damage and a lower microbial population. However, after 4 days, one log cycle increase in the total viable count and a 1.36 log cycle increase in the mold and yeast count occurred for the control-water sample. These values were much greater in the ultrasound-water sample (4 log cycles increase for both total viable/mold and yeast counts) after 4 days, which might be due to the mechanical damage that occurred in the fruits cell wall, facilitating the penetration of microbial cells. On the other hand, the damaged microbial cells had a chance to recover themselves during 4 days and then began to grow.

The lowest microbial population was observed in the NE and NE-ultrasound samples with a preference for the NE-ultrasound sample. Regarding the mold and yeast population, no significant differences were detected between these two samples, but NE-ultrasound exhibited better antimicrobial properties against total mesophilic bacterial count. The better NE-ultrasound function may relate to the cavitation effect produced by ultrasound which promoted the bacterial cell wall permeability and therefore, better penetration of TVO-NE into the bacterial cell. In addition, TVO bounds to DNA and alters DNA morphology^53^. TVO antimicrobial activity is improved by its nanoemulsification due to the small droplet sizes of NEs that facilitate the EO penetration and its homogeneous distribution^8^. Liu et al.^54^ demonstrated that the bacterial inactivation efficiency of photodynamic inactivation mediated by curcumin solid lipid nanoparticles was greater than that of free curcumin in carrot juice. Robledo et al.^55^ declared that coating strawberries with thymol-antimicrobial packaging decreased the fungal and yeast counts compared with the controls. He et al.^19^ reported that the combined use of ultrasound and thyme EO-NE indicated a synergistic antimicrobial effect against *E. coli*. Wang et al.^18^ also indicated that the application of ultrasound treatment in combination with cinnamon EO in fresh-cut asparagus efficiently reduced the microbial population to acceptable levels for 20 d. Jose et al.^56^ found that ultrasound could improve the efficacy of disinfectants. Wang et al.^18^ showed that ultrasonic-assisted citric acid and nisin washing immediately reduced the initial microbial load, and cinnamon EO treatment inhibited microbial growth during storage. Excellent bacteriostatic properties by ultrasound-assisted essential oil-loaded nanoemulsions pullulan-based film against *Escherichia coli* and *Staphylococcus aureus* were reported by Rashid et al.^57^ on strawberry fruit.

### Visual image and yellowing index

**Figure 5:**
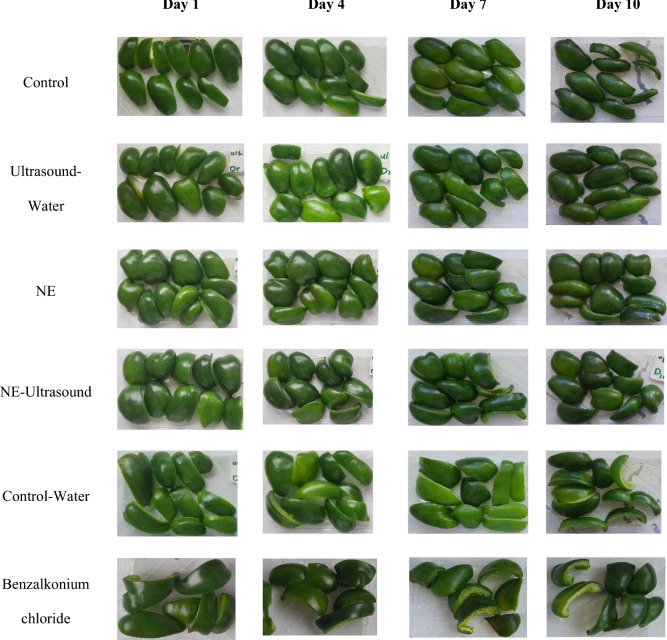
Visual image of different green bell pepper treatments during cold storage.

Figure 5 shows the bell pepper visual images during storage time. According to Fig. 5, the samples texture did not change until day 7; however, after that, the decay in the samples was distinguishable, which was more intense in the benzalkonium chloride sample, control, and ultrasound-water, respectively. The visual assessment of the samples showed the best quality for the NE-ultrasound and NE samples (p < 0.05). Although the control-water sample maintained the harness texture, it turned yellow with the extension of storage time. The color of some samples of ultrasound-water also changed to yellow which was lower than that of the control-water sample (p < 0.05).

**Figure 6:**
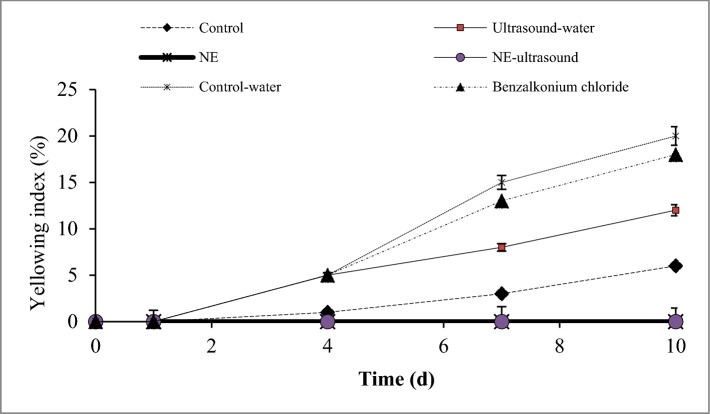
Yellowing index of different green bell pepper treatments during cold storage.

The yellowing index of fresh-cut bell pepper under different treatments is shown in Fig. 6. The yellowing index could reflect the yellowing degree of fresh-cut bell pepper. According to Fig. 6, the yellowing of fresh-cut bell pepper in the control-water, ultrasound-water, and benzalkonium chloride treatments became more intense with the extension of storage time. From day 4, some of the samples from these treatments turned yellow and then the yellowing index rose slowly. In this regard, control-water, ultrasound-water, benzalkonium chloride, and control treatments indicated the highest yellowing indexes, respectively (p < 0.05). After 10 days of storage, NE and NE-ultrasound treated samples remained green with the lowest yellowing index (about 0%).

The color change of fresh-cut bell pepper from green to yellow is mainly due to chlorophyll degradation. The results showed that NE and NE-ultrasound could effectively retard the degradation of chlorophyll and has the lowest yellowing index. Fresh-cut asparagus treated with ultrasonic-assisted citric acid and nisin washing combined with cinnamon EO fumigation (CEO) indicated the lowest yellowing index^18^.

## Conclusion

The findings presented in this paper demonstrate that the combination of TVO-NE and the ultrasound process was an effective decontamination tool, significantly reducing the microbial load on bell peppers. Meanwhile, according to the obtained results, treating green bell pepper with ultrasound in combination with *Thymus vulgaris* EO-NE reduced significantly the peroxidase activity, retained the antioxidant activity and green color as well as showed a strong antimicrobial and antifungal effect compared to other treatments. Although the experimental texture analysis did not show any significant effect due to ultrasonication, the electrolytic leakage was higher in this sample. However, the higher electrolytic leakage and weight loss of this sample did not have any adverse effect on the texture firmness, appearance, and visual color of the sample, which may be due to the lower respiration rate and peroxidase activity of the treatment. No mechanical damage or injuries were observed on the fruit surface or tissue. Therefore, ultrasound in combination with TVO-NE can be considered a feasible decontamination process. The antimicrobial/maintenance quality efficacy of the proposed technology on other kinds of fruits and vegetables as well as on inoculated samples is also important to be assessed in the future. It is essential to emphasize that any novel decontamination technology, such as the one presented here, must undergo validation and demonstration in a relevant industrial setting before it can be commercialized.

## Data Availability

The datasets used and/or analyzed during the current study available from the corresponding author on reasonable request.
